# Successful radiopeptide targeting of metastatic anaplastic meningioma: Case report

**DOI:** 10.1186/1748-717X-6-94

**Published:** 2011-08-12

**Authors:** Amir Sabet, Hojjat Ahmadzadehfar, Ulrich Herrlinger, Winfried Wilinek, Hans-Jürgen Biersack, Samer Ezziddin

**Affiliations:** 1 Department of Nuclear Medicine, University Hospital Bonn, Sigmund-Freud-Strasse 25, 53105 Bonn, Germany; 2 Department of Neurooncology, University Hospital Bonn, Sigmund-Freud-Strasse 25, 53105 Bonn, Germany; 3 Department of Radiology, University Hospital Bonn, Sigmund-Freud-Strasse 25, 53105 Bonn, Germany

**Keywords:** 18F-FDG-PET/CT, anaplastic meningioma, 111In-DTPA-octreotide, radio receptor therapy

## Abstract

A patient with anaplastic meningioma and lung metastases resistant to conventional treatment underwent radiopeptide therapy with 177Lu- DOTA-octreotate in our institute. The treatment resulted in significant improvement in patient's quality of life and inhibition of tumor progression. This case may eventually help to establish the value of radiopeptide therapy in patients with this rare condition.

## Background

Meningiomas are generally slow-growing lesions that arise from intracranial and spinal meninges. They are usually perceived as benign tumours for which radical surgery is the treatment of choice [1]. However, they may occasionally behave aggressively in atypical or malignant meningiomas, invading the brain and/or metastasising outside the CNS, which occurs in only 0.01% of all cases [2]. The most common extracranial location of metastasis is the lung followed by liver, lymph nodes and bones [3,4]. Meningiomas present ideal targets for somatostatin receptor scintigraphy (SRS) with 111In-DTPA-octreotide. However, the value of the radioreceptor therapy using radiolabeled somatostatin analog 177Lu-DOTA-octreotate is not yet well established in patients with metastasized or inoperable meningiomas [5,6]. Here, we present a patient with metastatic anaplastic meningioma who benefited from radiopeptide targeting.

## Case presentation

A 62 year old female with intracranial anaplastic meningioma was referred to our department for a restaging with 18F-fluorodeoxyglucose (FDG)-PET/CT. The patient suffered from a protrusio bulbi of the left eye and progressive facial pain. No conventional treatment option could be offered to the patient, who had undergone multiple surgical resections and percutaneous radiation before.

**Figure 1:**
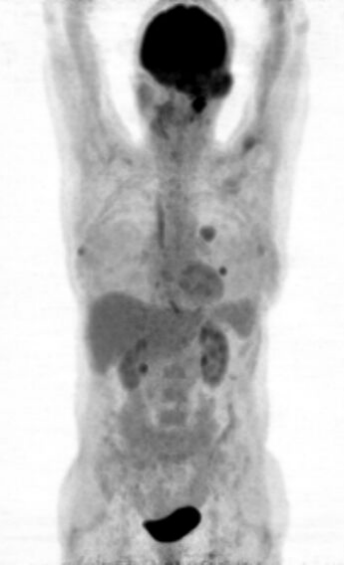
**Maximal intensity projection visualisation of PET/CT demonstrating the intracranial meningioma and its pulmonary metastases**.

**Figure 2:**
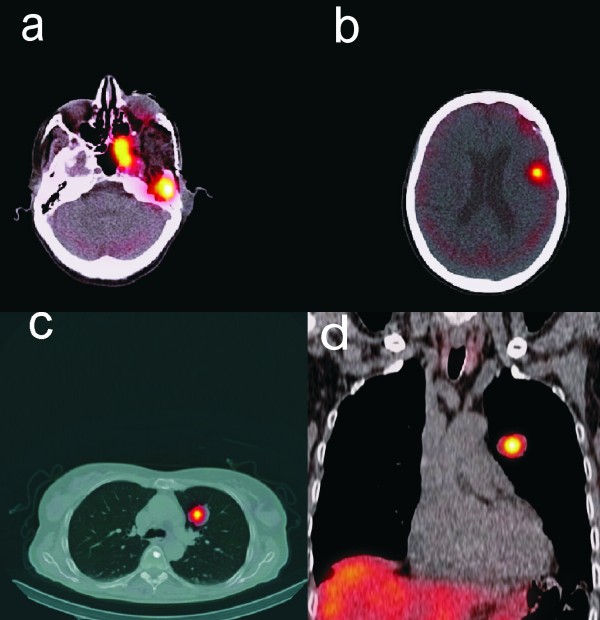
**SPECT/CT images of somatostatin receptor scintigraphy display avid uptake in the intracranial meningioma (2a and b) as well as in the pulmonary metastases (2c and d)**.

**Figure 3:**
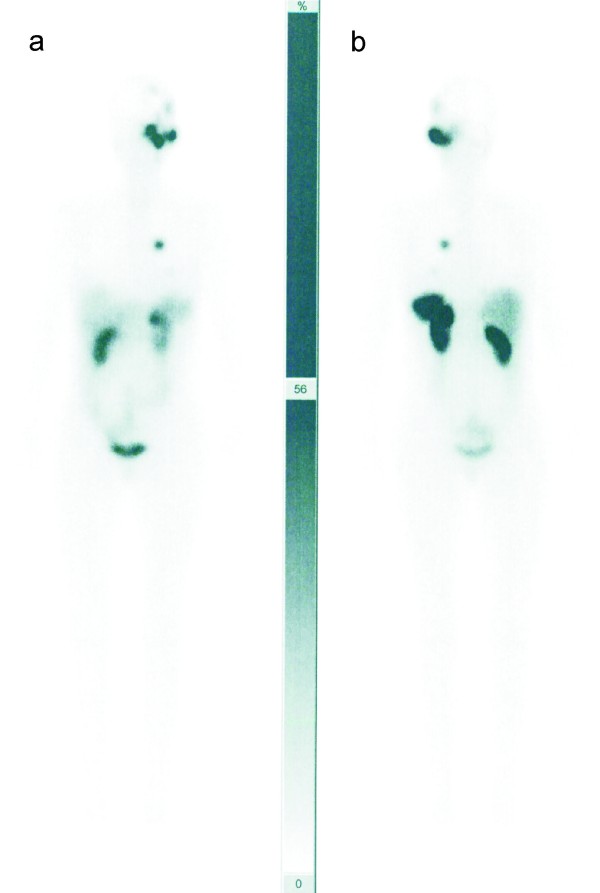
**Post-therapeutic 177Lu- DOTA-octreotate images show radiopeptide accumulation in the tumors (A: anterior view, B: posterior view)**.

The fused PET/CT images (Biograph; Siemens Medical Solutions Inc) manifested multifocal accumulation in the left temporal region with local bone infiltration. Furthermore, they demonstrated multiple pulmonary metastases in the upper lobe of the left lung (Figure 1). In view of these findings, including the diagnosis of pulmonary metastases, the patient was referred for SRS to evaluate the option of a palliative radiopeptide therapy with 177Lu- DOTA-octreotate. SRS images showed strong uptake in the left temporal region as well as in the upper lobe of the left lung, consistent with the PET/CT findings (Figure 2). Due to the abundance and high affinity of somatostatin receptors (sstr), we performed radiopeptide therapy with 177Lu- DOTA-octreotate consisting of 3 cycles (cumulative dose: 691 mCi) without any serious side effects (Figure 3). The patient experienced a dramatic reduction of facial pain assessed by visual analogue scale (VAS) as well as a significant improvement in quality of life with a 30% increase in her performance status using karnofsky scoring 6 weeks after commencement of the treatment. Disease stabilization could also be achieved, according to functional MD Anderson criteria, evaluated 3 months after termination of radiopeptide therapy [7]

## Discussion

The local recurrence rate of meningioma is determined by the extent of the resection, histopathological grade and biological aggressiveness of the tumor [8,9]. Once a meningioma recurs, it is more likely to recur again later, resulting in a poor prognosis of the patient [10]. 18F-FDG-PET/CT has been commonly used in patients with primary tumours of central nervous system including meningioma for tumor grading, determination of the prognosis and discrimination of tumor recurrence from radiation necrosis [11,12]. With their high sstr density and location outside the blood-brain barrier, meningiomas also present ideal targets for SRS with 111In-DTPA-octreotide which is the main imaging technique for neuro endocrine tuomors (NETs) but may be also used in other tumors expressing somatostatin receptors such as neuroblastoma, pheochromocytoma and paraganglioma [13-15]. This procedure is used apart from staging and monitoring the effect of treatment for selecting patients for peptide receptor radionuclide therapy (PRRT), primarily used in gastroenteropancreatic NETs with very encouraging results. The value of PRRT is not yet well established in patients with meningiomas [5]. Our patient experienced a dramatic symptomatic relief as well as a significant improvement in quality of life following the PRRT along with inhibition of tumor progression.

## Conclusions

The presented case may help to establish the value of PRRT in patients with the rare condition of anaplastic meningioma.

## Competing interests

The authors declare that they have no competing interests.

## Authors' contributions

Conception of the case report: A.S., S.E., HJ.B.; Collection and assembly of data: H.A., W.W., U.H.; Literature review and interpretation of data: A.S. S.E., H.A.; Drafting of the article: A.S., HJ.B., S.E.; Critical revision of the article for important intellectual content: U.H., H.A. W.W. All authors have read and approved the final manuscript.

## Consent

Written informed consent was obtained from the patient for publication of this Case report and any accompanying images. A copy of the written consent is available for review by the Editor-in-Chief of this journal.
